# Palliative care social work in medical institutions in mainland China: a qualitative study

**DOI:** 10.3389/fpubh.2026.1954058

**Published:** 2026-10-13

**Authors:** Wei Liu, Dan Li

**Affiliations:** School of Public Administration, Sichuan University, Chengdu, China

**Keywords:** health policy, mainland China, multidisciplinary collaboration, palliative care, qualitative research, social work

## Abstract

**Background:**

Palliative care in mainland China is expanding, but palliative social work remains unevenly defined and supported within medical institutions. How social workers negotiate patient, family, professional, organizational, and policy contexts remains insufficiently understood.

**Objective:**

To explore the experiences and institutional positioning of palliative social work in medical institutions in mainland China.

**Methods:**

A descriptive qualitative study was conducted in 11 medical institutions in Sichuan, Hebei, Guangdong, and Shanghai. Eligible institutions had provided palliative care for at least one year and employed full-time medical social workers. Semi-structured interviews were conducted with 39 participants, including 21 social workers, 5 physicians, 8 nurses, and 5 institutional managers. Data were analyzed in NVivo 12 using Braun and Clarke's reflexive thematic analysis with a primarily inductive orientation. Findings were organized across micro-, meso-, and macro-levels.

**Results:**

At the micro level, social workers supported person-centered end-of-life care by responding to multidimensional needs, mediating patient–family preferences, facilitating communication, and providing emotional, relational, and bereavement support. At the meso level, they acted as boundary-spanning coordinators across professional, institutional, hospital–community, and resource boundaries. At the macro level, their practice was constrained by uneven community resources, limited public understanding of palliative care, and gaps in policy, funding, staffing, training, supervision, career development, and professional standards. Although palliative social work was valued across stakeholder groups, its role remained inconsistently defined and insufficiently institutionalized. Social workers emphasized professional identity and role boundaries, managers highlighted staffing and financing, while clinicians focused more on coordination and public understanding.

**Conclusion:**

In the participating institutions, palliative social work contributed to relational care and cross-boundary coordination but lacked stable institutional support. Clearer role definitions, proactive referral pathways, multidisciplinary participation, adequate staffing and funding, professional training and supervision, and stronger hospital–community–home linkages are needed. Formal recognition and sustainable payment mechanisms warrant policy consideration, but their feasibility requires further research. These findings should not be generalized to all medical institutions in mainland China.

## Background

China is currently facing severe population aging. As the world's largest developing country, its aging process is characterized by the largest scale, rapid speed, and the unique and complex situation of “aging before getting rich” (1). The proportion of older adults deaths in the total population is constantly rising due to aging (2). Data from the National Health Commission and the Chinese Center for Disease Control and Prevention show that the number of deaths from chronic diseases in China has accounted for 88.5% of the total deaths of residents. Among the people over 65 in China, 75.8% have at least one chronic disease, and chronic diseases have become the “invisible killer” eroding the health of the older adults (3). According to data from the Ministry of Civil Affairs, there are currently about 35 million disabled older adults in China, accounting for 11.6% of all older adults (4). It is estimated that by 2035, the number of disabled older adults in China will reach 46 million. The older adults in China are facing serious health problems and low quality of death (5). This situation has created an urgent need for palliative care services in China. Although older adults make up a substantial share of patients receiving palliative care in China, this study did not restrict its focus to the care of older adults, and it examines palliative care social work across adult end-of-life care.

Palliative care, as a holistic care concept, aims to provide physical, psychological, and spiritual care and humanistic care for patients in the terminal stage of disease, control pain and discomfort symptoms, improve the quality of life, and help patients die peacefully and with dignity. Its core is “whole-person care” (6). Palliative care should be introduced at the early stage of the disease to achieve integration with conventional treatment plans (7). Palliative care was first introduced to China in the 1980s and was once called hospice care (8). The term “palliative care” began to be officially used in 2017 (9). According to the opinion of the National Health Commission, palliative care is for patients with life-threatening diseases and an estimated life expectancy of less than six months (10). While this policy definition is narrower than the broader international concept of palliative care introduced early in a disease trajectory (7), the term ānníng liáohù is the closest equivalent. For clarity and consistency with international literature, this paper will use the term ‘palliative care' while acknowledging the specific policy context in China. However, due to the taboo on death in Chinese culture, many terminally ill patients are reluctant to accept palliative care services, and many patients only receive palliative care in the last month of their lives (11).

Although China's palliative care services have made significant progress in recent years, there are still many problems with the quality of services (12). The “Quality of Death and Dying Index 2021” released by the Economist Intelligence Unit (EIU) ranked China 53rd among 81 countries and regions in terms of the quality and supply of palliative care in 2021 (13), reflecting that the development of palliative care in China is still in its early stage. The Chinese government has organized three batches of palliative care pilot projects in 2017, 2019, and 2023, respectively, promoting the establishment of a service system and achieving long-term development. Palliative care has officially risen from a professional field to a pilot policy of the national health system (14, 15). In 2025, the “Palliative Care Practice Guidelines (2025 Edition)” has been revised and officially released, marking a new stage of more standardized development of palliative care in China (10).

Palliative care social work refers to social workers using their professional skills to intervene in palliative care services, providing multi-dimensional services such as psychological, social, and spiritual support to terminally ill patients and their families, and conducting multi-disciplinary collaboration to improve the quality of life of terminally ill patients and their families (16). The core contradiction currently faced by palliative care in China is who will undertake non-medical needs (17). This contradiction cannot be resolved by the self-extension of the medical system but must introduce social work with psychological and social services as its core professional functions. The role and function of social work in palliative care are crucial, especially in maintaining the dignity of patients, alleviating physical and mental pain, and optimizing the family and social support system. The intervention of social work has significantly improved the quality of palliative care services (18). In recent years, with the release of relevant documents, palliative care social work in the mainland China has made significant progress. However, there are still deficiencies in terms of the number of professionals, systematic training, and professional identity (19). Culturally, the traditional concept of “cherishing life and fearing death” needs to be addressed (20). Operationally, there are issues such as team collaboration and the psychological pressure on social workers. Institutionally, there are challenges such as detailed standards, medical insurance payment, and cross-departmental collaboration (17). However, in general, the role of social workers in palliative care services is becoming increasingly important. Social workers are regarded as a vital force in expanding service supply and promoting fair and accessible services, and services are extending to communities and homes (21).

At present, there have been studies on palliative care social work services, but most of them focus on a certain aspect, such as the ethical dilemmas faced by social workers (22) or the content and importance of social worker training (23). In the mainland China, the main field for social workers to provide palliative care services is medical institutions (24). Existing research has not yet explored the experience of palliative care social work in medical institutions from an integrated perspective, including the necessity, functions, and challenges of conducting palliative care social work in medical institutions. Currently, the practice of palliative care social work in mainland China is still in the exploratory stage, and palliative care social work services still face many difficulties, all of which are worthy of study.

The purpose of this study is to explore the functions of social workers in palliative care in medical institutions in the mainland China, their necessity, and the challenges they face from the perspectives of social workers themselves and their multidisciplinary collaborators (physicians, nurses, and administrators). Ultimately, it is hoped that through a comprehensive understanding of social work services, comprehensive care can be provided to terminally ill patients, improving their quality of life and achieving high-quality development of palliative care services.

## Methods

### Design and settings

Using descriptive qualitative research, this study explores the experiences of palliative social work in Chinese medical institutions from the perspective of relevant practitioners (social workers, institution managers, doctors, nurses, etc.). Chinese medical institutions mainly consist of three levels: the first-level hospitals are mainly primary-level medical and health institutions, providing services to a certain population in a certain area, including community hospitals, township health centers, etc.; the second-level hospitals are generally regional hospitals, providing comprehensive medical services to multiple communities, undertaking teaching, research, and guiding lower-level hospitals, including county hospitals, district-level hospitals, etc.; the third-level hospitals are regional hospitals and above, providing high-level specialized services to several regions, undertaking higher-level teaching and research tasks, and being regional technical centers, including provincial-level and municipal-level major hospitals, and medical school-affiliated hospitals, etc.

This study was conducted in 3 provinces (Sichuan, Hebei, Guangdong) and 1 municipality (Shanghai) in mainland China, and the research scope covered the north and south, as well as the east and west of the mainland China (25).

### Participants

Through purposeful sampling, social workers and staff members of institutions working in medical facilities were selected. Firstly, contact the institution managers and introduce this research, including the research purpose and methods. After obtaining administrative approval, the researchers, under the guidance of the managers, went to the medical institutions for on-site individual in-depth interviews. The selection criteria for medical institutions included:

(1) having provided palliative care services for one year or more,
(2) having full-time medical social workers. This purposive sampling strategy was designed to select information-rich settings where palliative social work was relatively established, allowing for an in-depth exploration of its functions and challenges in practice. The qualification criteria for individual participants included:

(1) over 18 years old,
(2) having 1 year or more of experience in palliative care services,
(3) having relevant professional registration and career licenses. Non-Chinese participants were excluded. Although the primary focus of this study was on social workers' experiences, we intentionally included physicians, nurses, and administrators for three reasons. First, palliative care is delivered through multidisciplinary teams; understanding how other team members perceive and interact with social workers provides a more complete picture of social work practice. Second, the role and visibility of social workers in Chinese hospitals are often shaped by the attitudes of medical colleagues and institutional policies—perspectives that cannot be captured by interviewing social workers alone. Third, including multiple stakeholder groups allowed us to triangulate data and identify areas of convergence and divergence in perceptions of social work functions and challenges, thereby enhancing the credibility of our findings. Finally, this study interviewed 11 institutions in 4 provinces, including 3 primary hospitals, 4 secondary hospitals, and 4 tertiary hospitals. A total of 39 people participated in the interviews, specifically including 21 social workers, 5 doctors, 8 nurses, and 5 institution managers. The specific demographic information is presented in Table 1.

**Table 1:** Demographic characteristics of participants (*n* = 39).

Characteristic	Category	Social Workers (*n* = 21)	Physicians (*n* = 5)	Nurses (*n* = 8)	Administrators (*n* = 5)
Age (years)	Mean (range)	32.4 (24–51)	41.2 (35–52)	36.8 (29–48)	46.6 (39–58)
Gender	Male/Female	4/17	3/2	0/8	2/3
Years in palliative care	Mean (range)	4.2 (1–12)	8.6 (3–18)	6.3 (2–15)	5.8 (2–10)
Education	Bachelor/Master/Doctoral	15/6/0	0/2/3	6/2/0	2/2/1
Institution level	Primary/Secondary/Tertiary	4/8/9	1/2/2	2/3/3	1/2/2

### Interview guides

This study employed a self-developed interview guide, consisting of open-ended questions and non-guided questions. These guidelines were created based on literature research and policy studies, and were formulated through consultation with experts. The aim was to investigate the experiences of participants in the social work of palliative care in Chinese medical institutions, and to explore the current status of such social work in Chinese medical facilities.

The interview guides included six sections:

*(1) The current situation of palliative care services provided by institutions (such as the number of service personnel, beds, and the condition of the patients served)*
*(2) The situation of palliative care social workers (including the staffing situation and supporting facilities)*
*(3) The necessity of social work intervention in palliative care*
*(4) The roles and functions of social workers in palliative care*
*(5) The status of social workers' involvement in palliative care services, the difficulties and challenges faced*
*(6) The attitudes and intentions of patients and their families toward palliative care social work*.

Before the formal interviews, two institutions were selected, including 2 social workers and 2 medical staff, for a pilot study to assess the semantic clarity and cultural adaptability of the interview guide. Some participants showed misunderstandings about the social work of palliative care, which might disrupt the progress of the interviews. Based on the feedback, a question about “the attitudes of multidisciplinary team members such as doctors and nurses toward palliative care social workers” was added to facilitate more meaningful discussions. The 4 participants from the pilot testing were not included in the final sample and analysis.

### Data collection

The data were collected from September 2024 to October 2025. The research team was established before the research, consisting of university professors, graduate students, and undergraduate students from fields such as medicine, nursing, sociology, social work, and public administration. All team members had experience in qualitative exploratory research and received a one-week training on the concepts of palliative social work and interview skills before data collection. The interviews were conducted by two trained researchers with backgrounds in social work and nursing. Neither interviewer had any prior professional or personal relationship with the participants or the participating institutions. After the participants provided written informed consent, in-depth interviews were conducted. Throughout the interviews, techniques such as exploratory questions, clarification requests, and reflective listening were used to encourage participants to share their experiences and thoughts on the development of palliative social work in the context of the existing medical system (26). All interviews were recorded using smartphones or other devices, and the interviewees were informed of the study purpose and procedures and provided their consent in advance. A total of 39 individual in-depth interviews were conducted, with an average interview duration of 45 min (ranging from 30 to 75 min). The interviews were conducted by one or two researchers who spoke the local language to facilitate communication. The transcriptions of the recordings were converted into text. The researchers also recorded the non-verbal cues of the participants in real time, including facial expressions, gestures, and emotional reactions. To examine how the researchers' own disciplinary backgrounds and assumptions shaped data generation and interpretation, the interviewers wrote reflexive memos immediately after each interview and took part in regular team debriefings, in which emerging interpretations and the researchers' positioning were critically discussed. The interviewers' backgrounds in social work and nursing facilitated rapport with participants, while their status as outsiders to the participating institutions appeared to encourage participants to speak openly about institutional practices. The data collection was rigorous in accordance with the principles of credibility, reliability, confirmability, and transferability (27). Data collection and analysis proceeded iteratively. We assessed saturation in terms of 'meaning saturation,' stopping data collection when further interviews no longer generated new insights or dimensions to the developing thematic structure across the different stakeholder groups (28).

### Data analysis

Data analysis was guided by Braun and Clarke's reflexive thematic analysis (RTA) (29). We adopted a primarily inductive, data-driven orientation. Although the interview guide informed data generation, its domains were not used as a fixed coding framework; instead, codes and themes were actively developed through iterative engagement with the transcripts. The verbatim transcripts were imported into NVivo (Version 12) to facilitate data management and coding. The analysis began with data familiarization, followed by a systematic line-by-line initial coding by two authors. Subsequently, these initial codes were iteratively collated and interpreted to develop higher-order themes. For instance, descriptive codes such as “coordinating referrals with physicians and nurses,” “unclear role boundaries,” and “inconsistent referral pathways” were clustered into the integrated subtheme “Coordinating care across disciplines when role boundaries and referral pathways are unsettled.” Codes such as “limited recognition by the medical team” and “explaining social work expertise to healthcare professionals” informed the subtheme “Making social work visible and legitimate in multidisciplinary practice.” These related subthemes were then interpreted within the higher-order theme “Boundary-spanning coordination within multidisciplinary and organizational contexts.”

To enhance transcript accuracy, all proofread transcripts were returned to the respective participants for verification that their statements had been transcribed and represented faithfully. In the presentation of findings, participants are identified by codes to protect anonymity: institutional managers (M1–M5), social workers (S1–S21), doctors (D1–D5), and nurses (N1–N8).

After the thematic structure had been refined, coded accounts were compared across social workers, physicians, nurses, and institutional managers to identify convergence, divergence, and differences in emphasis concerning the value of palliative social work and barriers to its integration into medical institutions.

## Results

### Major themes

Data analysis identified three main themes and related subthemes, reflecting the experience of palliative social work at the micro, meso, and macro levels (Table 2).

**Table 2:** Themes and subthemes related to palliative care social work services.

Level	Theme	Subtheme
Micro: Patients and families	Relational negotiation of person-centered end-of-life care	1. Individualizing psychosocial care amid multidimensional and changing needs
		2. Mediating divergent preferences and communication around end-of-life care
		3. Sustaining emotional and relational support amid end-of-life distress
Meso: Healthcare teams and institutions	Boundary-spanning coordination within multidisciplinary and organizational contexts	1. Coordinating care across disciplines when role boundaries and referral pathways are unsettled
		2. Mobilizing institutional and community resources within fragmented care arrangements
		3. Making social work visible and legitimate in multidisciplinary practice
Macro: Policy, profession, and society	Positioning palliative social work within an incompletely institutionalized support environment	1. Extending hospital-based care into community networks amid uneven resource availability
		2. Shaping public understanding while navigating cultural discomfort and limited acceptance of death and palliative care
		3. Positioning the profession amid limited policy, funding, training, supervision, and career infrastructure

Following an initial inductive coding process, the resulting themes were organized using a micro-meso-macro ecological framework to interpret and structure the findings. This framework was not predetermined but emerged as a useful heuristic for understanding how palliative social work experiences were shaped across different levels of the system: interactions with patients and families (micro), multidisciplinary and organizational relationships (meso), and the broader policy and societal context (macro).

#### Theme 1: relational negotiation of person-centered end-of-life care

At the micro level, palliative social workers engaged directly with patients and families to address deeply personal, evolving, and often unspoken needs. Their work involved not only individualized assessment and support, but also the ongoing negotiation of communication, preferences, and emotional experiences within the intimate space of dying.

#### Subtheme 1: individualizing psychosocial care amid multidimensional and changing needs

Participants perceived that the needs of terminally ill patients are far beyond what can be covered by simple medical care. They are a complex and integrated entity that encompasses physical, psychological, social, and spiritual dimensions. Firstly, at the physiological level, in addition to pain and symptom control, patients often face a high degree of dependence on daily activities due to physical decline, which directly leads to the urgent need for long-term care resources, assistive devices, and the renovation of an aging-friendly environment. Secondly, in the psychological, social, and spiritual dimensions, patients commonly experience fear of death, anxiety about unfinished matters, questioning of life meaning, and intense feelings of loneliness. They also need to handle real-life pressures such as family relationships, property arrangements, and legal matters related to wills. Participants emphasized that patients and families often struggle to articulate their most pressing concerns, and that needs shift with disease progression, psychological adjustment, and family dynamics. Social workers therefore conducted comprehensive assessments of family systems, social support, economic status, and disease adaptation, and developed differentiated intervention plans tailored to each patient's evolving situation. This individualized psychosocial care required continuous reassessment rather than a one-time service provision.

“*Terminally ill patients actually face very complex needs. They are not only physical and medical, but also psychological and spiritual. Therefore, we cannot treat them as ordinary patients.” (N2)*

“*Because each patient's needs and subjective experiences during the service are different. If they don't express clearly, often what you provide is not what they need.” (S13)*

“*The same social work services have different demands in the elderly care aspect compared to the demands when patients are in the final stage and have their own cognitive abilities.” (S16)*

“We will also offer some psychological support and emotional counseling. Some cognitive aspects, such as adjustments to irrational thinking, etc. Through these professional methods of social workers, we will provide him with some psychological and emotional support.” (S5)

#### Subtheme 2: mediating divergent preferences and communication around end-of-life care

A recurring challenge at the micro level was the divergence between patient and family preferences concerning disclosure, treatment continuation, and end-of-life decisions. Participants described a recurring challenge how families often requested that diagnoses or prognoses be concealed from patients, while patients simultaneously sought truthful information; how patients sometimes wished to discontinue treatment while families insisted on aggressive intervention; and how requests such as euthanasia placed social workers in situations without clear resolution. Social workers navigated these tensions by mediating between respect for patient autonomy and preservation of family harmony, often serving as translators between medical language and family understanding, and facilitating family meetings to promote information exchange between patients, families, and clinicians.

“*Some patients who requested euthanasia told me that their condition was hopeless and that it would be better for their families to bear the burden and for society to save resources. They just wanted me to take the injection and get out of this quickly.” (S6)*

“*Some patients have multiple children, and their opinions often differ. A patient may request palliative care, but the children insist on continuing treatment, and this is when conflicts of opinion arise.”(N3)*

“*Many family members think that palliative care is just giving up treatment. Sometimes we have to explain to them that it's just a different approach focused on comfort. Otherwise, if we completely follow the family members' original wishes, it's very likely to result in excessive medical care.” (D5)*

#### Subtheme 3: sustaining emotional and relational support amid end-of-life distress

Beyond mediating communication, participants described social workers as providing sustained emotional and relational support to patients and families throughout the dying process and into bereavement. This included restoring social support networks, offering spiritual care, facilitating supportive and educational groups (such as stress management, death education, and grief counseling groups), and conducting home visits and follow-ups for special cases. Support also extended to bereavement care aligned with families' religious beliefs and cultural customs, including guidance on funeral procedures and grief counseling for surviving family members. This work required social workers to remain present with intense emotions over extended periods.

“*Every day, we conduct a comprehensive follow-up on each case of the families in the ward, helping them establish social connections and also facilitating the construction of the entire family relationship.” (S7)*

“*We will also from time to time set up some groups for them, such as stress management groups, supportive groups, these educational-style groups, and there are also these supportive groups that are not strictly therapeutic, like groups for death education and grief counseling. We will conduct these activities for this group of caregivers.” (S11)*

“*After a patient passes away, we provide bereavement follow-up counseling for the patient's family members, but mainly by phone and WeChat, with fewer home visits.”(S4)*

“*When entering the ward to face life, aging, and death, how to mentally prepare, how to pay attention to medical care, how to talk to patients about their conditions, and how to handle grief, these all have very high requirements for social workers.” (S3)*

### Theme 2: boundary-spanning coordination within multidisciplinary and organizational contexts

At the meso level, palliative social work was enacted across professional, organizational, and community boundaries. Social workers coordinated with healthcare professionals, connected patients and families with institutional and external resources, and worked to make their professional contribution visible within medical institutions.

#### Subtheme 1: coordinating care across disciplines when role boundaries and referral pathways are unsettled

Participants described multidisciplinary collaboration as necessary for responding to the multidimensional needs of patients at the end of life. Social workers worked with physicians, nurses, psychologists, and volunteers as part of a broader care network. However, the position of social workers within this collaborative process was not always clearly defined. Referral pathways and communication channels could be informal or dependent on whether patients and families explicitly requested help. These indicate that multidisciplinary collaboration was not simply a matter of adding social work to an existing team. It required the development of stable referral pathways, communication practices, and role boundaries. Where these arrangements were unsettled, social workers' participation in core care decisions remained limited.

“*At the hospital, we mainly work in collaboration with the medical team. The medical team is responsible for the part they are proficient in. Then, we, the social workers, psychologists, and volunteers, will work together to provide services for the terminally ill patients and their families for other needs - it's a model of multi-team collaboration.” (S18)*

“*Sometimes we actually don't know what the doctors said to the patients, nor do we know how much the patients received or understood. Our involvement in the core palliative care services is very limited.” (S16)*

“*If the patient requests social work services, we will sign an agreement and have the social workers follow up. If the patient doesn't express the need or doesn't say anything, we will not provide the service.” (M3)*

#### Subtheme 2: mobilizing institutional and community resources within fragmented care arrangements

Social workers mobilized resources both within institutions and across the hospital-community interface. Within institutions, they managed volunteer recruitment, training, and coordination, and provided psychological support to medical staff experiencing emotional exhaustion from ongoing exposure to death and dying. Across the hospital-community boundary, they attempted to link patients and families to community resources, welfare support, and external service providers. However, this mobilization occurred within highly fragmented care arrangements: institutional facilities for social work were often incomplete (sometimes lacking even an office), staffing ratios were extreme, and external resource systems lacked effective connection channels with hospital-based services.

“*Our social work department is responsible for the recruitment and training of social workers and volunteers, as well as the management of these activities. We regularly recruit some volunteers from the community. After training and review, they can then provide services in the palliative care wards.” (S6)*

“*We will provide support to our medical and nursing team. Because we have social work teachers with a background in psychology. If there are any emotional or psychological needs during the work of the doctors and nurses, we can also offer services.” (S9)*

“*We actually only have one position for social workers, and one person has to manage 400 people across the entire hospital. This actually creates a very difficult situation. Basically, social workers rarely come to ward.” (M1)*

“*We also don't have the environment to provide social worker services. When social workers arrive, they might not even have an office or any other facilities.” (M2)*

#### Subtheme 3: making social work visible and legitimate in multidisciplinary practice

Establishing the professional visibility and legitimacy of social work within medical institutions emerged as an ongoing challenge. Social workers were frequently misidentified as administrative staff or volunteers, both by medical colleagues and by patients and families. Their contributions were difficult to quantify in ways legible to medical hierarchies, and their humanistic and systemic perspectives were often marginalized in disease-centered decision-making. Even when institutional leaders recognized the value of social work and allocated positions from other departments, social workers still had to actively demonstrate their professional distinctiveness to be included in team decision-making. This paradox—being recognized as valuable yet remaining structurally marginal—was evident across participant accounts.

“*Many social workers nowadays do not have a proper identity. Some hospital social workers are employed by the hospital and thus become so-called administrative staff within the hospital. Therefore, we do not have a social worker identity in the hospital, but services cannot be halted. We can only explain to the patients that we are volunteers.” (S10)*

“*Many of the roles are different, but when providing services to individuals, the medical staff still have the most say. The value of our social workers is difficult to quantify and express.” (S9)*

“*Especially when frequently dealing with many terminally ill patients, they have to endure their negative emotions and family members' emotions. It is very likely to face the situation of emotional exhaustion.” (S6)*

“*Our positions are very scarce, but we still allocated a position from another department to the social workers. This way, it will be more convenient for us to carry out our services.” (M2)*

“*I think social workers are extremely important because we are usually very busy and many psychological and social support services cannot be provided. The coordination work done by social workers between patients and their families can significantly reduce our stress.” (D2)*

### Theme 3: positioning palliative social work within an incompletely institutionalized support environment

At the macro level, palliative social work was shaped by a broader support environment. The profession's development was shaped by uneven resource availability across regions, persistent cultural resistance to open discussion of death, and the absence of a coherent policy and professional infrastructure.

#### Subtheme 1: extending hospital-based care into community networks amid uneven resource availability

Social workers sought to extend palliative care beyond hospital walls by building connections with community organizations, conducting community health screenings, and linking patients and families to external resources. Collaborations with community centers, women's federations, and local partners enabled activities such as free medical check-ups, health education, and community-based palliative care promotion. However, resource availability was highly uneven: urban settings offered richer supplementary resources, while rural areas required social workers to provide more essential, life-sustaining assistance with fewer resources. Because social workers were institutionally based within hospitals, developing genuine community-based practice remained structurally constrained.

“*There are some collaborations with the social workers in the neighborhood. For instance, when we carry out some public welfare activities, these community-based activities. Or in our current projects, there is health education and death education related to palliative care, which are linked with the neighborhood. The social workers from the Women's Federation will also have some coordination and cooperation during the activities.” (S11)*

“*Sometimes we contact the community to see if we can conduct free medical check-ups. Last year, our medical check-up activities were all over the planned quota. The community publicity efforts were also mostly beyond the planned limit.” (S13)*

“*The service locations of domestic social workers are mainly within hospitals. They spend their entire working week at the hospital. So how can the attribute of social service be demonstrated by them?” (S11)*

“*In urban areas, social work may involve more resources, but the service recipients in the city are more of the supplementary type. However, for the service recipients in rural areas, it is a life-saving assistance.” (S15)*

#### Subtheme 2: shaping public understanding while navigating cultural discomfort and limited acceptance of death and palliative care

Participants described limited public understanding and acceptance of palliative care. Some healthcare professionals framed palliative care as a different approach to death and dying, rather than as the abandonment of treatment. However, families and members of the public could interpret palliative care as giving up. In response, social workers and healthcare institutions used public education and community activities to promote understanding of death, dying, and palliative care. These activities included death cafés, reading groups, and educational sessions. Life education was also conducted through creative and participatory approaches. Thus, social workers contributed to shaping public understanding by explaining the meaning of palliative care and creating opportunities for public discussion of death and dying. The findings suggest that this work took place within a cultural context in which death was often difficult to discuss and palliative care was not yet widely understood.

“*Most people still cannot accept palliative care. They believe that palliative care means giving up and is a passive state.”(S10)*

“*Many members of the public do not accept or understand the concept of palliative care. Previously, in our country, it was called hospice care. However, there might be many misunderstandings among the public. Therefore, since 2017, our country has started to call it palliative care, and our department is also called the Palliative Care Ward.” (N7)*

“*We conduct various activities such as death cafes, reading clubs, and all kinds of learning sessions. These activities are open to patients, their families, or even healthy people. They all come to participate in the services. This is a kind of social promotional service.” (S1)*

“*Life education is essentially a matter of establishing a belief, specifically a belief about life and death. Last year, we made some attempts. For instance, we explored the way of performing death and observing death through drama, using the method where each person tells their own story. Additionally, we also considered how to approach death in film.” (S3)*

#### Subtheme 3: positioning the profession amid limited policy, funding, training, supervision, and career infrastructure

The development of palliative social work as a profession was constrained by an incomplete institutional infrastructure at the macro level. Participants described the absence of mandatory national policies requiring social work positions in medical institutions, the lack of dedicated staffing budgets and reimbursement mechanisms, and the absence of professional title sequences or promotion pathways for hospital-based social workers. National-level service standards specifically for palliative social work were largely absent, with only scattered provisions and significant regional variation. Industry-level professional standards for ethics, service processes, and performance evaluation still need to be improved. These structural gaps meant that even where individual institutions and professionals recognized the value of social work, systemic support for career development, supervision, and sustainable service delivery remained inadequate.

“*The biggest challenge for our healthcare system in providing palliative care is the lack of policy at the community health centers. We could have social workers or have several social workers assigned to ensure the numbers are accurate. The government will cover the costs once the numbers are confirmed because currently there is no corresponding confirmation. This might be the biggest challenge we are facing at this stage.” (M5)*

“*I think the main difficulty lies in the policies. Currently, there is no policy requiring the establishment of dedicated social worker positions, no positions, no salaries. How can social worker services be implemented?” (M2)*

“*Now there are many service standards for palliative care, but these standards do not specifically mention the standards for social workers. It feels like social workers are an embedded and supplementary element, and they have never broken away from the medical and nursing orientation within the traditional hospital system.” (S7)*

“*At present, we only have job responsibilities within the organization, but our region has not established a very complete set of service standards and procedures.” (S8)*

“*Especially in the field of medical social work, the lack of continuing education and professional supervision is even more severe among palliative care social workers. Because as social workers working in the medical field, we not only need professional social work knowledge, but also need the support of medical and psychological knowledge. However, our learning of the latter two is limited to textbook knowledge, and we lack hands-on practice and training during internships.” (M3)*

### Cross-stakeholder synthesis: convergence and divergence in the positioning of palliative social work

A comparison across stakeholder groups revealed broad agreement that biomedical care alone could not fully address the multidimensional needs of patients and families at the end of life. However, the groups framed the core value of palliative social work through different professional and organizational lenses. Social workers primarily described their contribution as a distinctive form of relational and psychosocial practice, involving the identification of unspoken needs, mediation of patient–family communication, emotional support, and the coordination of formal and informal resources. Physicians tended to frame social work as extending care into areas that exceeded the time or scope of medical practice and as reducing the coordination and emotional burden placed on clinical staff (D1, D2). Nurses emphasized the multidimensional nature of patients' needs and the need to improve public understanding of palliative care (N2, N7). Institutional managers more often evaluated social work in operational terms, including its capacity to address matters that medical staff could not manage and to improve service procedures and organizational functioning (M4). Thus, although the stakeholder groups converged on the need for social work, they evaluated its value against different reference points: professional psychosocial expertise, complementarity to clinical care, holistic responsiveness, and organizational effectiveness.

The differences between stakeholder perspectives were particularly apparent in accounts of multidisciplinary integration. Social workers foregrounded uncertain professional identity, limited access to clinical information, difficulty demonstrating their contribution within medically dominated hierarchies, and restricted participation in core care decisions (S9, S10, S16). Institutional managers, by contrast, tended to describe integration in terms of formal service pathways, staffing arrangements, and organizational authorization. For example, M3 described a request-led pathway in which social work services were initiated only when patients explicitly asked for them. When considered alongside S16′s account of limited access to doctor–patient communication and restricted involvement in core palliative care, these perspectives reveal an important difference in how the same organizational arrangement may be understood. What appears administratively to be a workable referral procedure may be experienced by social workers as a reactive pathway that limits early involvement and may overlook needs that patients and families are unable or unwilling to articulate. Physicians' recognition of social workers' value in reducing clinical workload (D2) further indicates that interpersonal recognition of social work did not consistently translate into formalized referral pathways, routine information sharing, or decision-making authority.

Stakeholders also differed in the barriers they emphasized. Institutional managers focused primarily on the structural feasibility of service provision, including the absence of designated positions, inadequate staffing, insufficient facilities, unclear salary or funding mechanisms, and limited policy support (M1, M2, M5). Social workers focused more strongly on the professional consequences of these structural conditions: being classified as administrative personnel or volunteers, having their contributions treated as difficult to quantify, lacking clearly defined role boundaries, and working without sufficiently developed service standards (S7–S10). Physicians and nurses more often emphasized barriers encountered at the clinical and public interface, including limited time for non-medical support, family misconceptions that palliative care meant abandoning treatment, and limited public understanding of death and palliative care (D1, D5, N7). These perspectives were not necessarily contradictory; rather, they located the barriers to social work integration at different points within the same institutional system.

Taken together, the cross-stakeholder comparison suggests that the integration of palliative social work cannot be achieved solely by demonstrating its usefulness in individual cases. It also requires the translation of cross-professional recognition into stable referral pathways, access to relevant clinical communication, clearly defined professional roles, designated staffing and funding arrangements, and institutional and professional standards. The multi-stakeholder accounts therefore position the development of palliative social work as both a relational-professional process and an organizational-structural process.

## Discussion

### Overview and conceptual contribution

This study examined how social workers and their multidisciplinary collaborators experience the delivery of palliative social work in mainland Chinese medical institutions. International scholarship has repeatedly established that social workers make an essential contribution to palliative care by addressing psychological, social, and spiritual needs that biomedical care cannot reach (18, 30, 31). Our analysis offers two interpretive contributions. The first is a structural paradox: palliative social work was consistently recognized as valuable across all four stakeholder groups, yet remained institutionally marginal in role definition, staffing, decision-making authority, and professional infrastructure. The second concerns the nature of the social work contribution itself, which is better specified as boundary spanning (32, 33) than as the delivery of discrete services. Read through an ecological lens (34), the three themes are therefore not parallel categories but three levels of one mutually constitutive system.

### Recognized value amid institutional marginalization

The tension at the center of our data is that social work was valued interpersonally but marginalized institutionally. Physicians and nurses valued social workers for extending care beyond the reach of medical practice and for absorbing relational labor that clinical work could not contain (D2); administrators valued them for improving service procedures and organizational functioning (M4). Yet the same stakeholders described social work as reactive and request-led, and as situated outside core clinical decision-making (M3; S16). Social workers experienced this as a failure of professional identity: they were absorbed into administrative categories, misrecognized as volunteers, and unable to have their contribution quantified within a medically dominated hierarchy (S9, S10).

The coexistence of recognition and marginalization is not peculiar to China. Studies of interprofessional teams in other health systems have documented that social workers are valued for psychosocial expertise while being treated as auxiliary to medical decision-making, and that unclear role boundaries oblige psychosocial professionals to continually reassert their legitimacy (16, 35, 36). Research on multidisciplinary team meetings similarly shows that these ostensibly collaborative routines can reproduce medical dominance rather than dissolve it (17). Our findings offer one interpretation of why recognition may not translate into institutional positioning. Following Abbott (37), a profession's standing depends on securing jurisdiction (recognized control over a task domain) and on the institutional arrangements that sustain it. Palliative social work in China claims no such jurisdiction: medical social work is itself emergent, lacks a licensing monopoly and a career title sequence, and depends on institutional goodwill rather than mandatory staffing. Where jurisdiction is absent, recognition is easy to grant because it costs nothing; it remains discursively available while being structurally unsecured. This is the dynamic Gieryn (38) termed boundary work, in which a profession's claims to competence must be continually performed rather than assumed.

This interpretation is reinforced by the fact that marginalization persisted in precisely the settings we selected because they were information-rich and had full-time social workers. These findings suggest that the problem may be structural rather than solely attributable to under-resourcing. Recognition is a necessary but insufficient condition for integration: unless it is translated into staffed positions, defined referral pathways, access to clinical information, and participation rights in decision-making, it carries no institutional force. China's policy environment sharpens this problem. Although national guidelines and pilot programmes have advanced palliative care provision (39, 40), the establishment of social work posts remains encouraged rather than mandated, and reimbursement, continuing education and supervision mechanisms for palliative social work remain underdeveloped (19, 21).

### Boundary spanning as the distinctive function of palliative social work

The second contribution concerns how we conceptualize what palliative social workers actually do. Their role cannot be adequately described as the delivery of bounded services; it is better understood as boundary spanning across the micro, meso and macro levels of the care system. Boundary spanners are actors who facilitate transactions and the flow of information between people or groups that have no direct access to one another, or no basis on which to trust one another (33). The concept captures the work we observed more faithfully than the language of “roles” or “functions”: at the micro level, social workers moved between the patient's inner world and the family system, translating between what patients could articulate and what families could accept, and mediating divergent preferences about disclosure and treatment (S6; D5). At the meso level, they moved across professional and organizational boundaries. They linked physicians, nurses, psychologists and volunteers, bridging the hospital–community interface, and mobilizing welfare, legal and material resources (S18; M1). At the macro level, they acted as translators between institution and society, promoting public understanding of death and palliative care while interpreting cultural values for clinical practice (S1, S3). The service categories identified in earlier analyses of palliative social work, including services to patients and families, to institutions, and to society (18, 41), are thus the micro-, meso- and macro-level expressions of a single boundary-spanning function.

Framing these activities as boundary spanning also explains why social work is simultaneously indispensable and vulnerable. Boundary spanners are structurally necessary precisely because they operate in the gaps between established domains (32, 42); by the same token, their work maps onto no single jurisdiction, which makes it difficult to codify, evaluate and reward within existing professional and organizational structures. This allows us to reinterpret findings that would otherwise read as an inventory of “challenges.” Unclear role boundaries, limited access to clinical communication, weak referral pathways and the absence of palliative-social-work-specific service standards are not incidental regrets but the predictable organizational corollaries of a boundary-spanning role that has not been institutionally recognized as such. The implication is that strengthening palliative social work cannot rest on exhortations about its importance; it requires building the structures through which boundary-spanning work can be stabilized (e.g., referral protocols, information-sharing norms, shared decision-making rights, and codified competencies) (23, 30). Where these are absent, as our inter-stakeholder comparison showed, administrators may regard a request-led referral arrangement as workable while social workers experience the same arrangement as reactive, and physicians' interpersonal appreciation of social work does not translate into routine information sharing.

### A balanced cultural interpretation: culture as resource as well as constraint

Cultural taboo surrounding death, filial expectations and family-centered decision-making did produce recurring tensions: family members concealed diagnoses from patients, insisted on continued treatment, and interpreted palliative care as abandonment (N3; D5; S10); the tendency to defer referral until the final month of life compounds these pressures (11). Yet the same cultural resources that generated these tensions also supplied the relational infrastructure on which social workers depended. Families were, in most cases, the primary source of practical and emotional care. Filial commitment, however demanding, sustained a level of involvement that no formal system could replace; and the indigenous ideal of a “good death” offered a shared vocabulary through which social workers, patients and families could negotiate goals of care (S3; S11). This is consistent with evidence that cultural values in Chinese palliative care are not simply obstacles but organizing frameworks for how dying is understood and managed (20, 43).

### The interdependence of levels

Read across ecological levels, our findings describe a mutually constitutive system rather than three parallel themes. Macro-level gaps in policy, funding and professional standards shape the meso-level organizational arrangements that determine access to referrals, information and decision-making. Those arrangements, in turn, set the conditions under which micro-level psychosocial care is delivered: whether social workers can identify unspoken needs early, whether they are included in conversations about prognosis, and whether they can accompany patients and families through bereavement. Institutional theory helps specify the mechanism: organizations adopt practices that confer legitimacy in their environment, and where the institutional environment provides no template for social work, its presence remains ceremonial rather than consequential (44). This has a direct methodological implication for intervention: efforts confined to one level are unlikely to succeed. Training individual social workers without reforming structures, or creating positions without defining roles and pathways, will reproduce the paradox of recognized value and institutional marginalization.

### Public health implications

For policy, the analysis suggests several options that warrant consideration and evaluation. These include transitioning palliative social work from an “encouraged” to a more institutionalized role through mechanisms such as designated positions within palliative care services, dedicated funding and payment mechanisms, inclusion of social work indicators in service quality evaluation, and national standards defining scope of practice, competencies, and supervision requirements. For institutions, it argues for constructing the connective tissue of boundary spanning including stable referral pathways, routine clinical communication channels, and participation in multidisciplinary decision-making, rather than the mere presence of social workers on the payroll. For education and professional development, it highlights the case for training in death education, communication with dying patients, family-centered decision support and ethical decision-making, together with post-qualification supervision capable of sustaining practitioners exposed to continuous loss (23, 30). Finally, the cultural analysis suggests that strengthening public and professional understanding of palliative care should be framed not as a campaign against culture but as a dialogue with it, drawing on indigenous ideals of a good death to support, rather than replace, medical and psychosocial care.

### Strengths and limitations

This study explores the experience of palliative social work services in medical institutions from the perspective of mainland China, revealing the actual practice and evolving logic of social work services under the interweaving of the medical system, resources, and cultural concepts. Three themes were discovered: the experience of palliative social work at the micro, meso, and macro levels. The study's strengths include its multicenter design and multidisciplinary participant sample. However, this research still has the following limitations: First, the institutional eligibility criteria may have resulted in the inclusion of relatively experienced, information-rich, and potentially better-resourced settings. Participating institutions had provided palliative care services for at least one year and had full-time medical social workers. Therefore, they may differ from institutions without established palliative care services or dedicated social work positions. Although the sample included institutions from eastern and western parts of mainland China and participants from several professional groups, it could not capture the full geographical, institutional, and resource diversity of mainland China. The findings should therefore not be generalized to all medical institutions in mainland China. Secondly, some research subjects were recruited through personal connections of the researchers, which may have introduced selection bias toward more experienced or better-resourced institutions and participants with more favorable views of social work, thereby limiting the transferability of the findings. Third, patients and family members were not included as interview participants. Accordingly, statements about patients' or families' needs, understanding, preferences, and attitudes represent second-order accounts based on the observations and interpretations of social workers, healthcare professionals, and institutional managers, rather than direct accounts of patients or family members. In the future, research on palliative social workers can consider involving social workers from more extensive regions, especially focusing on social workers and related medical staff providing palliative care in communities and at home. Additionally, incorporating the viewpoints of patients and their families would help provide a more comprehensive understanding of the necessity, functions, and obstacles of palliative social workers.

## Conclusion

This study indicates that, in the 11 participating medical institutions in Sichuan, Hebei, Guangdong, and Shanghai, palliative social work was widely viewed as valuable but was not yet consistently institutionalized. At the micro level, social workers supported the relational negotiation of person-centered end-of-life care by responding to patients' and families' multidimensional and changing needs, mediating divergent preferences, facilitating communication, and providing emotional, relational, and bereavement support. At the meso level, they acted as boundary-spanning coordinators who connected patients and families with healthcare professionals, institutional and community resources, and other forms of psychosocial support. At the macro level, however, their work was shaped by uneven community resources, limited public understanding of palliative care, and gaps in policy, funding, training, supervision, career development, and professional standards.

The findings suggest that the integration of palliative social work requires more than demonstrating its usefulness in individual cases. In the participating institutions and comparable settings, medical institutions should clarify the scope and boundaries of social work practice, establish proactive and needs-based referral pathways, improve routine communication between social workers and clinical teams, and include social workers in relevant multidisciplinary discussions and decision-making processes. Adequate staffing, appropriate workspaces, professional training, clinical supervision, and career development opportunities are also needed. Stronger links between hospital-based, community-based, and home-based services may further improve continuity of support, particularly where community resources are unevenly distributed.

At the policy and system levels, participants' reports of the absence of designated posts, dedicated funding, reimbursement mechanisms, and clear professional standards provide an empirical basis for considering more formal recognition of palliative social work within medical institutions. This may include evaluating the feasibility of regulatory requirements for social work positions and sustainable payment arrangements, including whether eligible palliative social work services could be supported through existing medical-insurance or other public financing mechanisms. However, this qualitative study did not assess the legal, financial, or implementation feasibility of these policy options. Therefore, such measures should be adapted to local institutional conditions and evaluated through further policy and implementation research rather than assumed to be universally applicable.

A culturally responsive approach should be family-inclusive while remaining responsive to the preferences and rights of individual patients. Family-centered decision-making and culturally specific understandings of a “good death” may create tensions concerning disclosure, autonomy, and treatment preferences, but they may also provide relational resources for communication, caregiving, reconciliation, meaning-making, and bereavement support.

Because the sample was restricted to medical institutions that had provided palliative care for at least one year and employed full-time medical social workers, these findings are most applicable to relatively established or information-rich settings. They should not be generalized to all medical institutions across mainland China. Further research should include patients and family members directly and should examine palliative social work in community and home-based settings, as well as in institutions with less developed palliative care services.

## Data Availability

The datasets presented in this article are not readily available because de-identified transcripts are not publicly available because contextual details may remain potentially identifiable, but that relevant data may be available from the corresponding author upon reasonable request and subject to ethical restrictions. Requests to access the datasets should be directed to DL, ldan99@126.com.
